# Notes and News

**Published:** 1896-06-27

**Authors:** 


### Notes and News.

(Collected and Communicated.)

The trustees of Smith's Charity have granted £100 to the Royal Westminster Ophthalmic Hospital.

Dr. W. J. Collins, M.S., B.Sc.Lond., F.R.C.S., has been appointed full surgeon to the Royal Eye Hospital, Southwark.

Mr. Henry Harben has sent a donation of £500 to the North London Hospital for Consumption, Mount Vernon, Hampstead.

The students of pharmacy in Paris have formed themselves into an association, of which M. Berger has accepted the presidency.

The London County Council have purchased a site for a seventh County Asylum. The new asylum will be situated at Epsom. The land purchased consists of over 1,000 acres of park-like land, much of which is let on lease. The cost of the property was £40,000.

In mentioning Sir William McCormack as a likely successor to Mr. Christopher Heath in the presidency of the Royal College of Surgeons, an American contemporary describes him as one of the most hidebound of conservatives, and as likely to resist the introduction of any reform.

Up to the time of our going to press £31,500 has been received as the result of the Hospital Sunday in the metropolis this year. The largest amounts have been collected at St. Michael's Church, Chester Square (£1,508); Christ Church, Lancaster Gate (£1,305); the parish of St. Mary Abbott's, Kensington (£531); St. Paul's, Onslow Square (£510); and All Saints, Ennismore Gardens (£502). The fund, in addition, has received a legacy of £500 from Mr. J. Smith, £312 from the Gresham Trustees, and £200 from "Delta."

The Hospital Saturday collection at Birmingham has already passed the sum asked for this year, as it now amounts to over £14,000.

The lesson enforced by the experience of unfortunate Gloucester is still having effect, and many local authorities are making inquiries into the vaccination returns in their areas. In the metropolis it is alarming to learn that the percentage of unvaccinated is very large, reaching amongst the children of Hackney to no less than 41 per cent.

A most attractive fête was held this week at Hanworth Park, the residence of Mr. Lafone, M.P., in aid of Guy's Hospital. The fête was opened by the Duchess of Wellington, and the arrangements were very complete. The scene was rendered specially picturesque by the erection of wooden structures representing the architecture of different countries. The entertainments were numerous and varied.

The new Cottage Hospital at Tilbury was opened by Mr. Passmore Edwards on the 21st inst. The site was given by the London and India Docks Joint Committee, the hospital by Mr. Passmore Edwards, who contributed £2,000, whilst many trades unions and organisations have made liberal donations. The opening was the occasion of a large demonstration in the neighbourhood, 3,000 attending the ceremony.

Bedford is to have a new county hospital. The plans have been selected and approved. The cost is calculated at about £26,000, or 7s. the cubic foot. The general idea of the building is an administrative block, with wards having south aspect connected by corridors. The building is to be of red brick with stone facings. Messrs. Stephen Salter and Adams, of Woburn Place, are the architects.

The visit paid by the Princess of Wales to St. Mary's Hospital on Monday gave very great pleasure to the inmates and all concerned in the working of the hospital. H.R.H. was accompanied by the Princesses Victoria and Maud, and was attended by Miss Knollys, who is always actively associated in the kindly deeds of the Princess of Wales. Sir William Broadbent received the Royal visitors, who visited all the wards, and spoke to the patients in each.

It is very evident that Irish workmen are learning to appreciate the advantages of hospital treatment. Quite recently the working men's committee of the Frome Hospital in Belfast visited the institution to specially bring forward its claim for support, and it is stated that no less than £2,400 has been collected by them in aid of the medical charities, this large amount having been collected nearly entirely in coppers.

The Northern Hospital at Liverpool has been the recipient of a useful gift in the shape of a new ambulance. The ambulance service at this hospital is one of the features of the institution, and the new carriage generously presented to the hospital by Mr. Councillor Louis Cohen is a valuable addition to the resources of the hospital, being of the same pattern as that constructed for the use of the St. John Ambulance Society in London.

